# Sodium-glucose Co-transporter 2 (SGLT2) inhibitor dapagliflozin acutely activates cardiomyocyte HIF-1α signaling via succinate, a signaling metabolite

**DOI:** 10.1016/j.jphs.2026.01.008

**Published:** 2026-04

**Authors:** Tatsuyuki Sato, Takayuki Isagawa, Yuki Sugiura, Daigo Sawaki, Yu Nakagama, Takahiro Kuchimaru, Shun Minatsuki, Shigeru Sato, Kazutoshi Ono, Ariunbold Chuluun-Erdene, Hiroaki Semba, Masamichi Ito, Toshinaru Kawakami, Ryohei Tanaka, Masaya Sakamoto, Masataka Asagiri, Hiroshi Harada, Christian Stockmann, Tomo Yonezawa, Yasushi Hirota, Yasutoshi Kido, Kinya Otsu, Takahide Kohro, Ichiro Manabe, Issei Komuro, Norihiko Takeda

**Affiliations:** a Division of Bioconvergence, Center for Molecular Medicine, Jichi Medical University, Tochigi, Japan; b Department of Cardiovascular Medicine, Graduate School of Medicine, The University of Tokyo, Tokyo, Japan; c Research Fellow of the Japan Society for the Promotion of Science, Japan; d Data Science Center, Jichi Medical University, Tochigi, Japan; e Department of Biochemistry, Keio University School of Medicine, Tokyo, Japan; f Division of Clinical Pharmacology, Department of Pharmacology, Jichi Medical University, Tochigi, Japan; g Department of Virology & Parasitology, Research Center for Infectious Disease Sciences, Graduate School of Medicine, Osaka Metropolitan University, Osaka, Japan; h Division of Nephrology, Department of Internal Medicine, Jichi Medical University, Tochigi, Japan; i Department of Diabetes, Metabolism and Endocrinology, School of Medicine, International University of Health and Welfare, Mita Hospital, Tokyo, Japan; j Department of Pharmacology, Yamaguchi University Graduate School of Medicine, Yamaguchi, Japan; k Laboratory of Cancer Cell Biology, Graduate School of Biostudies, Kyoto University, Kyoto, Japan; l Institute of Anatomy, University of Zurich, Switzerland; m Cancer Research Center Zurich, Winterthurerstrasse 190, CH-8057, Zurich, Switzerland; n Division of Functional Genomics and Therapeutic Innovation, Research Center for Advanced Genomics, Graduate School of Biomedical Sciences, Nagasaki University, Nagasaki, Japan; o Department of Obstetrics and Gynecology, Graduate School of Medicine, The University of Tokyo, Tokyo, Japan; p The School of Cardiovascular Medicine and Sciences, King's College London British Heart Foundation Centre of Excellence, London, United Kingdom; q National Cerebral and Cardiovascular Center, Suita, Japan; r Department of Systems Medicine, Graduate School of Medicine, Chiba University, Chiba, Japan; s Department of Frontier Cardiovascular Science, The University of Tokyo, Tokyo, Japan; t International University of Health and Welfare, Chiba, Japan

## Abstract

SGLT2 inhibitors are widely used to treat patients with chronic heart failure, and several studies have shown that the efficacy of SGLT2 inhibitors also extends to acute heart failure. However, the mechanisms remain unknown. Here, using knockout mice and pharmacological approaches, we show that short-term SGLT2 inhibitor treatment activates hypoxia-inducible factor-1α (HIF-1α) signaling in cardiomyocytes, and further pharmacological studies raised the possibility that this effect is mediated by ketone body-derived succinate. One week of Dapagliflozin administration upregulated the expression of HIF-1α target genes, and the effect was abolished in cardiomyocyte-specific HIF-1α knockout mice. Metabolome analysis and enzyme-based assays revealed that, following one week of short-term Dapagliflozin treatment, ketone body levels in the heart increased, leading to an accumulation of succinate, which may act as a signaling metabolite that stabilizes HIF-1α. Administration of pimozide, which is a succinyl-CoA:3-ketoacid CoA transferase (SCOT) inhibitor that inhibits ketone body metabolism, abolished dapagliflozin-elicited activation of HIF-1α signaling. These results, although not conclusive, can be plausibly explained if short-term Dapagliflozin treatment activates HIF-1α signaling in cardiomyocytes via ketone body-derived succinate. Our study raises the possibility that HIF-1α plays a role in the effects of SGLT2 inhibitors and highlights HIF-1α as a speculative target for future studies.

## 1 Introduction

Sodium-glucose co-transporter 2 (SGLT2) inhibitors have emerged as a novel standard therapy for patients with chronic heart failure.^1^, ^2^, ^3^ It is now known that SGLT2 inhibitors are effective not only for HFrEF (heart failure with reduced ejection fraction) but also for HFpEF (heart failure with preserved ejection fraction),^4^^,^^5^ and guidelines and consensus reports now include SGLT2 inhibitors as one of the first-line treatments for both types of chronic heart failure^6^, ^7^, ^8^. More recently, multiple studies have been ongoing on acute heart failure, with some showing beneficial effects even in the acute phase.^9^, ^10^, ^11^, ^12^, ^13^ However, the mechanisms by which SGLT2 inhibitors have an effect on acute pressure overload remain unknown.

Hypoxia-inducible factor-1α (HIF-1α) is a key transcriptional regulator of cell metabolism. Under normoxic conditions, HIF-1α is hydroxylated by HIF-prolyl hydroxylases (HIF-PHs), which target it for degradation. HIF-1α is stabilized in hypoxia and induces the expression of glycolytic enzymes, as well as *vascular endothelial growth factor-a* (*Vegfa*), a key regulator of angiogenesis. We previously showed that the HIF-1α signal increases capillary density in the heart and contributes to the maintenance of cardiac homeostasis.^14^^,^^15^ It should also be noted that, as excessive activation of HIF-1α signaling can impair cardiac function, precise control of HIF-1α signaling is essential for maintaining cardiac function.^16^

SGLT2 inhibitors not only reduce blood glucose levels but also exert multiple metabolic effects in target organs.^17^, ^18^ In the heart, SGLT2 inhibitors change metabolic fuels from glucose to fatty acids or ketone bodies.^18^, ^19^ During ketone body oxidation, succinyl-CoA:3-ketoacid CoA transferase (SCOT) transfers coenzyme-A (Co-A) from succinyl-CoA to acetoacetate and forms acetoacetyl-CoA and succinate. Notably, succinate can suppress HIF-PHs and activate HIF-1α signaling, and thus it is termed a signaling metabolite.^20^ The link, however, between SGLT2 inhibitors and HIF-1α has not been elucidated.

Here, we adopted a murine model of acute pressure overload and evaluated the short-term effects of dapagliflozin, an SGLT2 inhibitor, on cardiac function and metabolic signaling. Dapagliflozin activated cardiomyocyte HIF-1α signaling, and, although its functional consequences are inconclusive in the current study, this may be relevant to the cardioprotective effect of SGLT2 inhibitors. Mechanistic analysis raises the possibility that SCOT-mediated ketone body oxidation increased intracellular succinate levels in dapagliflozin-treated hearts, leading to the activation of HIF-1α signaling. The results highlight a potential role of cardiomyocyte HIF-1α signaling during treatment with SGLT2 inhibitors.

## 2 Methods

### 2.1 Mice and animal studies

Cardiomyocyte-specific HIF-1α deficient mice were generated using B6.129-Hif1a^tm3Rsjo^/J (HIF-1α^flox/flox^, JAX Stock #007561, The Jackson Laboratory) and Tg(Myh6-cre)1Jmk (αMHC-Cre).^21^^,^^22^ Cre-negative littermate mice were used for control. Wild-type C57BL/6J mice were purchased from CLEA Japan (Tokyo, Japan). Mice were housed in a specific pathogen-free facility with a 12-h light/12-h dark cycle. Mice aged 7–12 weeks were used for the experiment. An anesthetic combination of medetomidine hydrochloride (30 μg/mL), midazolam (0.4 mg/mL), and butorphanol tartrate (0.5 mg/mL) dissolved in sterile saline was used for the surgical procedures.

The transverse aortic constriction (TAC) operation was performed as follows. First, mice were anesthetized with an intraperitoneal injection of the anesthetic combination (10 μL/g body weight). After anesthesia, mice were fixed on a heated plate in the supine position. The chest was opened via a sternotomy, and the transverse aorta was exposed. A 27-gauge needle was placed next to the aortic arch, the suture was tied firmly by a 7-0 silk ligature around the 27-gauge needle together with the aorta, and the needle was removed. After chest closure, a midazolam antagonist, atipamezole, was administered intraperitoneally (1.2 μg/g body weight), and the mice were observed until they fully recovered. Cardiac function was evaluated using the Vevo2100® Ultrasound System (FujiFilm VisualSonics, Toronto, Canada). To quantify the degree of constriction, the peak aortic flow velocity was measured using pulsed wave Doppler at the point of constriction, and the pressure gradient was calculated using the modified Bernoulli equation. Following the TAC procedure, mice with an ejection fraction of less than 80% were selected for the analyses.

All animal experiments were approved by the University of Tokyo Ethics Committee for Animal Experiments and the Use and Care of Experimental Animals Committee of Jichi Medical University. The procedures strictly adhered to the guidelines for animal experiments of the University of Tokyo and Jichi Medical University. Dapagliflozin was provided by AstraZeneca (Cambridge, UK) or purchased from Sigma (SML2804; Sigma-Aldrich, Burlington, MA, USA). Dapagliflozin was dissolved in drinking water (8 μg/mL) and given to the mice *ad libitum*. Pimozide (Sigma-Aldrich, Burlington, MA, USA) was dissolved in corn oil and administered via oral gavage every 2–3 days.

### 2.2 RNA isolation, reverse transcription, and quantitative real-time polymerase chain reaction (qPCR)

Total RNA from mouse hearts was isolated using the NucleoSpin RNA kit (Takara Bio, Shiga, Japan). Complementary DNA was synthesized from RNA template using ReverTra Ace reverse transcriptase (TOYOBO Co., Ltd, Osaka, Japan). Quantitative PCR was performed using the Roche LightCycler 480 with THUNDERBIRD SYBR qPCR Mix (TOYOBO Co., Ltd) and the relative standard curve method. Results were normalized to *Glucuronidase Beta*(*Gusb*) expression. The sequences of the primers are described in Table 1.

**Table 1:** List of primers used for quantitative PCR analyses.

Primer	Sequence
*Gusb* Forward	5′-AAAATCACCCTGCGGTTGT-3′
*Gusb* Reverse	5′-TGTGGGTGATCAGCGTCTT-3′
*Ldha* Forward	5′-CTTAAGGAAGAGCAGGCTCCC-3′
*Ldha* Reverse	5′-TCTCGCCCTTGAGTTTGTCT-3′
*Nppa* Forward	5′-CCTAAGCCCTTGTGGTGTGT-3′
*Nppa* Reverse	5′-CAGAGTGGGAGAGGCAAGAC-3′
*Nppb* Forward	5′-GCTGCTTTGGGCACAAGATAG -3′
*Nppb* Reverse	5′-GCAGCCAGGAGGTCTTCCTA-3′
*Pgk1* Forward	5′-CTGTGGTACTGAGAGCAGCAAGA-3′
*Pgk1* Reverse	5′-CAGGACCATTCCAAACAATCTG-3′
*Slc2a1* Forward	5′-CTGGACGACGGACCCTGC-3′
*Slc2a1* Reverse	5′-AAAGAAGGCCACAAAGCCAAAG-3′
*Vegfa* Forward	5′-GGAGAGCAGAAGTCCCATGA-3′
*Vegfa* Reverse	5′-ACTCCAGGGCTTCATCGTTA-3′
*Pdh1* Forward	5′-GGCAACTACGTCATCAATGG-3′
*Pdh1* Reverse	5′-GATTGTCAACATGCCTCACG-3′
*Pdh2* Forward	5′-CATGAAGTACAGCCAGCATAC-3′
*Pdh2* Reverse	5′-TCTCGCTCGCTCATCTGCA-3′
*Pdh3* Forward	5′-TGTCTGGTACTTCGATGCTGA-3′
*Pdh3* Reverse	5′-TCTTTAGCAAGAGCAGATTCAGTTT-3′

### 2.3 Tissue sampling and metabolome analysis by ion chromatography (IC)-mass spectrometry (MS)

Metabolome analyses were performed as previously described.^23^ The detailed method is described in the supplementary methods. In brief, hydrophilic metabolites were extracted from the mice's hearts using a laboratory microwave instrument (MMW-05; Muromachi Kikai, Tokyo, Japan) and a manual homogenizer (Finger Masher, AM79330; Sarstedt, Tokyo, Japan). For internal control, L-Methionine sulfone, 2-Morpholinoethanesulfonic acid, monohydrate [MES], D-Camphor-10-sulfonic Acid Sodium Salt [CSA], 3-Aminopyrrolidine, and Trimesate were used. Anionic metabolites were measured using an Orbitrap-type MS (Q-Exactive focus; Thermo Fisher Scientific) connected to a high-performance IC system (ICS-5000+, Thermo Fisher Scientific). Compound Discoverer 3.1 (Thermo Fisher Scientific) and MetaboAnalyst (v6.0)^24^ were used for analyses.

### 2.4 Blood and tissue metabolite measurement

Whole blood glucose and β-hydroxybutyrate levels were quantified using Glucocard G Black (Arkray, Kyoto, Japan) and FreeStyle Libre (Abbott, Maidenhead, UK), respectively. The mitochondria fraction of cardiac tissues was isolated using the Mitochondria Isolation Kit for Tissue (Thermo Fisher Scientific, Waltham, MA, USA) according to the manufacturer's instructions. Protein concentration was quantified using Pierce Rapid Gold BCA Protein Assay Kit (Thermo Fisher Scientific). Succinate and α-ketoglutarate levels were quantified using the Succinate Assay Kit (Abcam, Cambridge, UK) and the Alpha-Ketoglutarate Assay Kit (Abcam). Absorbance was measured using an iMark microplate reader (Bio-Rad, Hercules, CA, USA). Luminescence was measured with an EnVision 2104 Multilabel Reader (PerkinElmer, Waltham, MA, USA). Mitochondrial succinate and α-ketoglutarate levels were normalized to mitochondrial protein abundance.

### 2.5 Histological study

Formalin-fixed paraffin-embedded (FFPE) heart sections were used for the histological analyses. To evaluate vessel density, the FFPE sections were stained using biotinylated isolectin-B4 (Vector Laboratories Inc., Burlingame, CA, USA) and streptavidin-conjugated FITC (Vector Laboratories Inc.). The number of vessels was evaluated in three randomly selected fields per slice and averaged. Fluorescent images were acquired using an FSX-100 inverted microscope (OLYMPUS, Tokyo, Japan). Sirius Red and Fast Green stains were performed to visualize tissue fibrosis. Bright-field images were acquired using the virtual slide system VS120 (OLYMPUS). The fibrotic area was calculated compared to the total tissue area. All the images were analyzed using Image J (version 2.1.0) software (NIH, Bethesda, MD, USA).

To evaluate the frequency of HIF-1α-positive cardiomyocytes, the FFPE sections were stained using an anti-HIF1α antibody (NB100-134; Novus Biologicals, CO, USA), and the bright field images were acquired using BZ-X800 (KEYENCE, Osaka, Japan). The positive frequency of HIF-1α-positive cardiomyocytes was manually counted in five randomly selected fields per slice using Image J software (NIH).

### 2.6 Statistical analyses

Data are expressed as the mean ± standard deviation (SD). The statistical significance of differences was tested with the Student's t-test or two-way Analysis of Variance (ANOVA) and Holm-Šidák's multiple comparisons test. *P*-values less than 0.05 were considered statistically significant. The outlier detection was performed by robust regression and the outlier removal (ROUT) method.^25^ Statistical analyses and outlier detection were performed with GraphPad Prism 10.2.2 (GraphPad Software, San Diego, CA, USA).

## 3 Results

### 3.1 Cardiomyocyte HIF-1α knockout mice showed a decrease in left ventricular ejection fraction in the context of dapagliflozin treatment

**Fig. 1:**
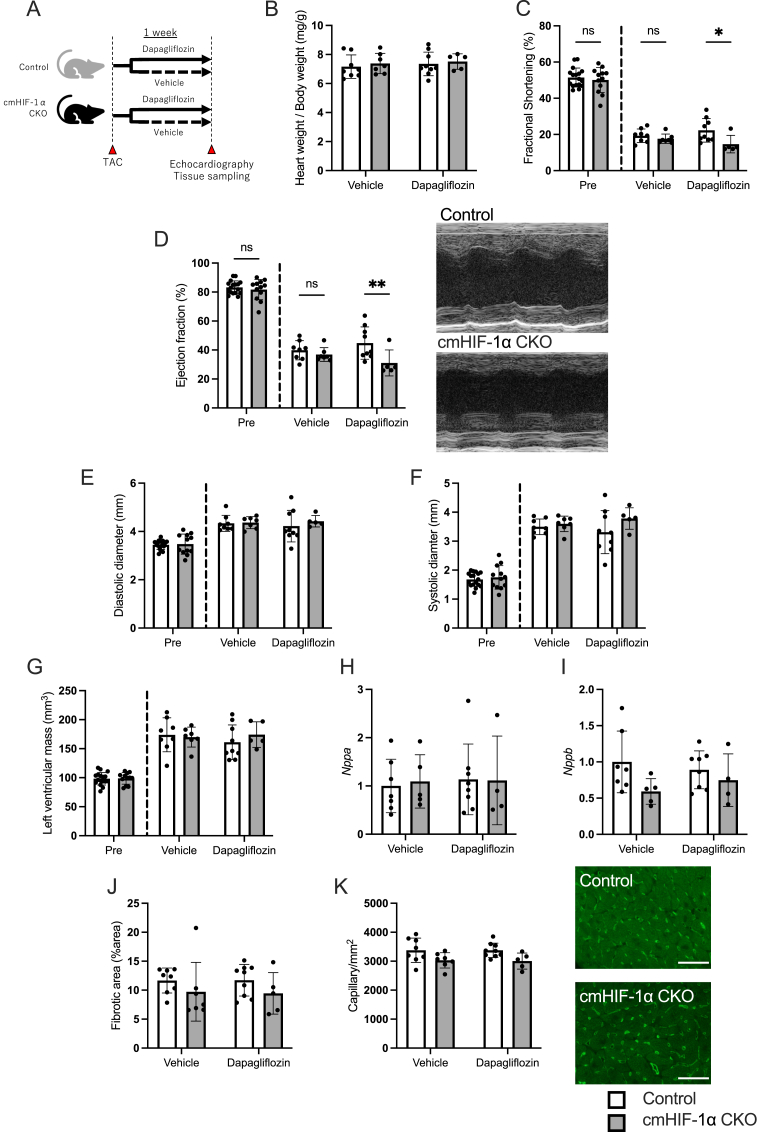
Context-dependent roles of cardiomyocyte HIF-1α signaling in cardiac function following pressure overload with or without dapagliflozin **(A)** Scheme of the experiment. Control mice and cardiomyocyte-specific hypoxia inducible factor-1α knockout (cmHIF-1α CKO) mice were subjected to transverse aortic constriction (TAC). Dapagliflozin or vehicle was dissolved in drinking water and given to the mice *ad libitum* starting from the day of surgery. Data show heart weight normalized to body weight **(B)**, cardiac function evaluated using echocardiography **(C**–**G)**, cardiac tissue transcript levels of genes related to cardiac remodeling **(H, I)**, fibrotic area **(J)**, and capillary density **(K)** evaluated 1 week after TAC surgery. Transcript levels were normalized to *Glucuronidase beta* (*Gusb*). Scale bar = 50 μm. Results are expressed as mean ± SD. N = 4–9 per group. Two-way Analysis of Variance (ANOVA) and Holm-Šidák's multiple comparisons test were used to compare the differences between groups.

TAC is a commonly used animal model in which acute pressure overload to the left ventricle elicits a cardiac hypertrophic response and a decrease in systolic function. In this study, we administered dapagliflozin to TAC-operated mice for 1 week and observed its acute effects on cardiac function. To dissect the roles of cardiomyocyte HIF-1α signaling in relation to SGLT2 inhibition, we administered dapagliflozin to TAC-treated cmHIF-1α CKO and the control mice (Fig. 1A).

The heart weight of cmHIF-1α CKO was comparable to that of the control mice (Fig. 1B). The knockout of cardiomyocyte HIF-1α did not affect cardiac function following one week of TAC in vehicle-treated mice. In contrast, the knockout of cardiomyocyte HIF-1α led to decreased ejection fraction and fractional shortening in dapagliflozin-treated mice, indicating that cardiomyocyte HIF-1α signaling is augmented in dapagliflozin-treated mice (Fig. 1C and D). There was no significant difference in the left ventricular mass, diastolic diameter, and systolic diameter (Fig. 1E–G). There was no significant difference in the transcript levels of genes related to cardiac remodeling, including *natriuretic peptide type A* (*Nppa*) or *natriuretic peptide type B* (*Nppb*) (Fig. 1H and I). Dapagliflozin did not affect the extent of cardiac fibrosis or capillary density in either group (Fig. 1J and K). After we obtained the data, we noticed that there could be potential outliers that could potentially undermine the actual relationship. Therefore, we performed a post-hoc outlier test for Fig. 1H–J using the ROUT method. The test detected no outliers in Fig. 1H and I, but one outlier in Fig. 1J. When the value was excluded and the statistical analysis was re-performed, the knockout of cardiomyocyte HIF-1α showed a significant decrease in fibrosis in vehicle-treated mice, but not in dapagliflozin-treated mice (Supplementary Fig. 1). The degree of TAC was equivalent between cmHIF-1α CKO and the control mice (Supplementary Fig. 2). The knockout efficiency evaluated within the whole heart extract by qPCR was similar between vehicle- and dapagliflozin-treated mice (Supplementary Fig. 3). The results are inconclusive as to whether HIF-1α signaling contributes to the preservation of cardiac function in the dapagliflozin-treated mice. However, the difference in the response of cardiac function to HIF-1α knockout in vehicle- and dapagliflozin-treated mice prompted us to hypothesize that dapagliflozin administration activates cardiomyocyte HIF-1α signaling.

### 3.2 Dapagliflozin induced HIF-1α accumulation in TAC-operated mice

**Fig. 2:**
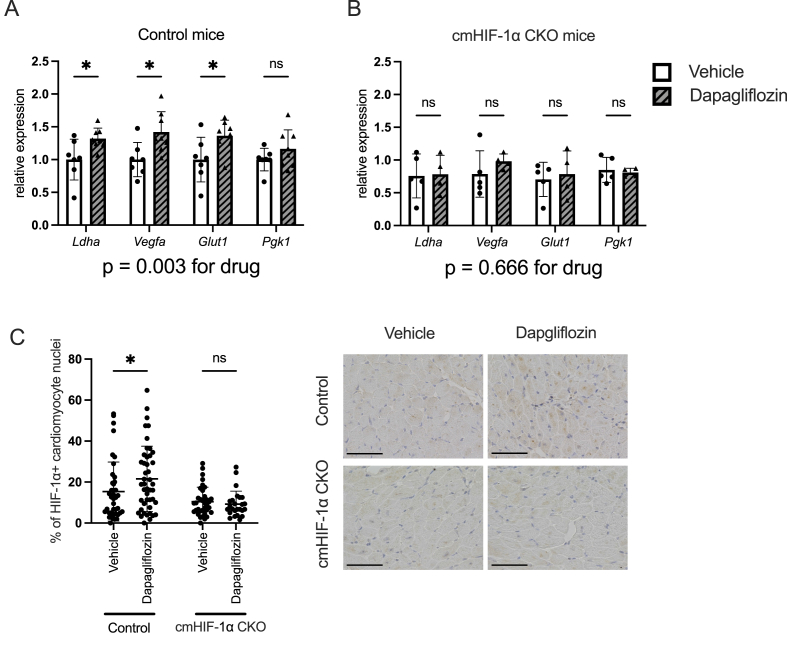
Dapagliflozin increased HIF-1α target gene expression in the TAC model **(A, B)** Transcript levels of hypoxia inducible factor-1α (HIF-1α) target genes, *lactate dehydrogenase A* (*Ldha*), *phosphoglycerate kinase 1* (*Pgk1*), *solute carrier family 2, member 1* (*Slc2a1*), and *vascular endothelial growth factor-a* (*Vegfa*), were analyzed in the hearts sampled after 1 week of dapagliflozin administration in control and cardiomyocyte-specific HIF-1α knockout (cmHIF-1α CKO) mice. N = 4–8 per group. Transcript levels were normalized to *Glucuronidase beta* (*Gusb*). **(C)** Quantitative analysis and representative images of immunohistochemistry of HIF-1α following 1 week of transverse aortic constriction (TAC) and dapagliflozin treatment. A total of 25–45 slides per group from 5 to 9 mice per group were used for analysis. Results are expressed as mean ± SD. Scale bar = 50 μm. Two-way Analysis of Variance (ANOVA) and Holm-Šidák's multiple comparisons test were used to compare the differences between groups.

To test this hypothesis, we measured the transcript levels of canonical HIF-1α target genes, including *lactate dehydrogenase A* (*Ldha*), *phosphoglycerate kinase 1* (*Pgk1*), *solute carrier family 2, member 1* (*Slc2a1*), and *Vegfa*. In the hearts of control mice, dapagliflozin significantly upregulated the expression of this set of genes. However, the expression of the set of HIF-1α target genes remained unchanged in dapagliflozin-treated cmHIF-1α CKO hearts (Fig. 2A and B, Supplementary Table 1). Immunohistochemistry showed that the dapagliflozin-treated mice hearts have a higher number of HIF-1α-positive cardiomyocytes compared to vehicle-treated mice hearts (Fig. 2C). In cmHIF-1α CKO mice, we did not detect any difference in the number of HIF-1α-positive cardiomyocytes in vehicle- and dapagliflozin-treated hearts (Fig. 2C). The expression levels of HIF-PHs, which target HIF-1α, remained unchanged (Supplementary Fig. 4). These results indicate that dapagliflozin stabilizes HIF-1α protein and activates cardiomyocyte HIF-1α signaling and its downstream targets with a mechanism that is independent of HIF-PH expression. The upregulation of HIF-1α target genes was no longer observed in the mice treated for 4 weeks, suggesting that dapagliflozin-induced activation of HIF-1α is a temporal effect (Supplementary Fig. 5).

### 3.3 Dapagliflozin administration accelerates ketone body oxidation in the murine heart

**Fig. 3:**
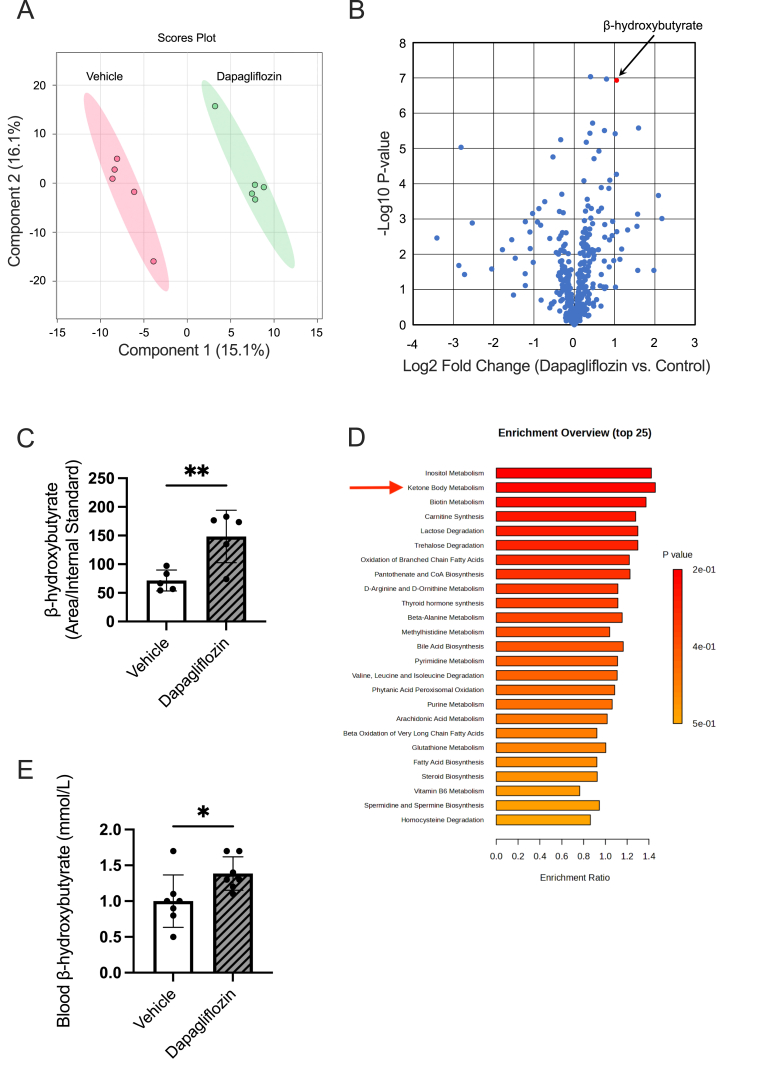
Dapagliflozin administration accelerates ketone body oxidation in the murine heart **(A)** Partial least squares-discriminant analysis (PLS-DA) of the hydrophilic metabolites extracted from the hearts following 1 week of dapagliflozin or vehicle administration. Each point represents the result of an individual mouse. The primary principal component and the secondary principal component are displayed on the x-axis and y-axis, respectively. N = 5 per group. **(B)** Volcano plot analysis of the differential metabolites between dapagliflozin and vehicle-treated mice. The x-axis shows the log_2_ fold change (dapagliflozin vs. vehicle), whereas the y-axis shows the log_10_ p-value of the difference between dapagliflozin- and vehicle-treated mice. **(C)** Levels of β-hydroxybutyrate in the heart following 1 week of dapagliflozin administration. N = 5 per group. **(D)** The overview of enriched metabolites in the heart following 1 week of dapagliflozin administration. Pathways are displayed in order of increasing p-value, and the x-axis shows the enrichment ratio. **(E)** Blood β-hydroxybutyrate level was measured following 1 week of dapagliflozin administration. N = 7 per group. Results are expressed as mean ± SD. The Student's t-test was used to compare the differences between the two groups.

To understand the link between dapagliflozin and HIF-1α signaling, we performed a non-targeted metabolomics analysis of heart tissues in wild-type mice. Partial least squares-discriminant analysis (PLS-DA) revealed significantly distinct metabolic profiles in dapagliflozin-treated mice (Fig. 3A). Among them, β-hydroxybutyrate, a ketone body, accumulated significantly, and the ketone body metabolite sets were enriched in dapagliflozin-treated hearts (Fig. 3B–D), while other metabolites in the tricarboxylic acid cycle, pentose-phosphate pathway, and nucleotide synthesis pathway were unchanged (Supplementary Fig. 6–9). The blood level of β-hydroxybutyrate was also elevated in dapagliflozin-administered mice (Fig. 3E), suggesting that dapagliflozin administration activates ketone body oxidation in the murine heart. Acetoacetate, another type of ketone body, showed no difference between dapagliflozin- and vehicle-treated groups (Supplementary Fig. 7).

### 3.4 Succinate accumulates in dapagliflozin-treated mice hearts and activates cardiac HIF-1α signaling

When hydroxylating the HIF-1α protein, HIF-PHs utilize molecular oxygen and α-ketoglutarate as co-substrates and generate succinate as a byproduct. Hence, succinate accumulation in turn can suppress HIF-PHs through product inhibition and activate HIF-1α signaling in a hypoxia-independent manner.^26^ Notably, succinate is synthesized during ketone body oxidation, where SCOT transfers Co-A from succinyl-CoA to acetoacetate, raising the possibility that succinate may act as a mechanistic link between ketone body oxidation and HIF-1α.

**Fig. 4:**
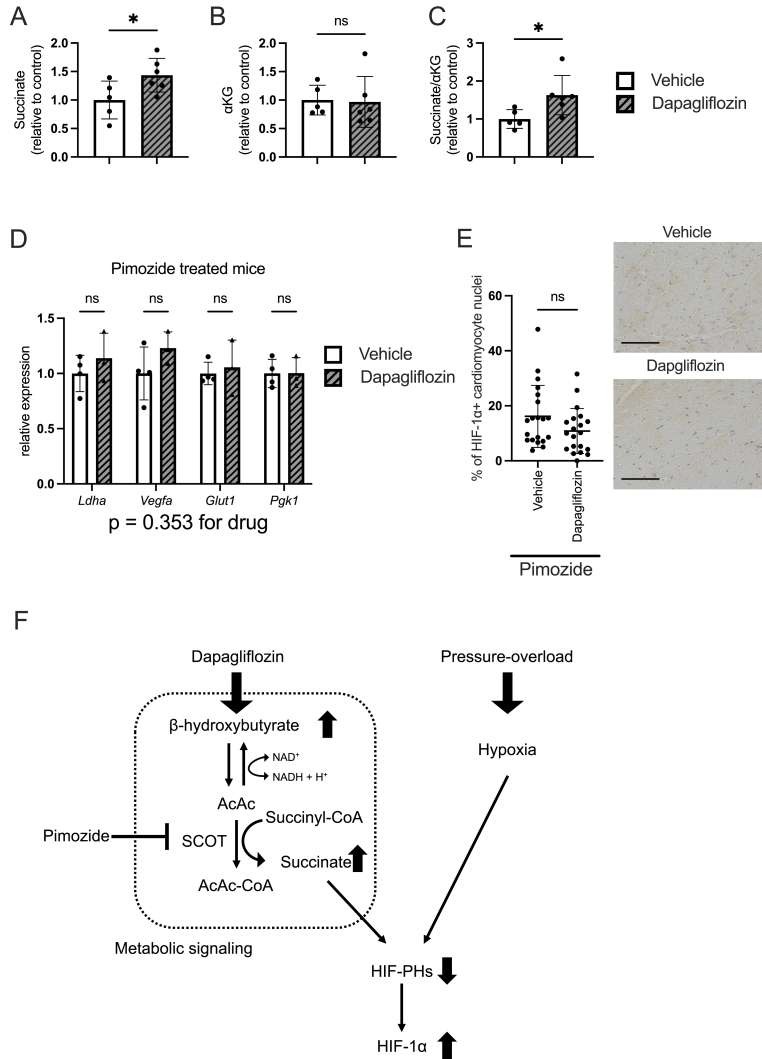
Succinate accumulates in the dapagliflozin-treated heart and activates HIF-1α signaling **(A–C)** Succinate and α-ketoglutarate levels in the mitochondrial fraction of cardiac tissues were quantified 1 week after dapagliflozin or vehicle administration. N = 5–6 per group. Results are expressed as mean ± SD. The Student's t-test was used to compare the difference between the two groups. **(D)** Pimozide was administered every 2–3 days to transverse aortic constriction (TAC)-operated mice, and the transcript levels of *lactate dehydrogenase A* (*Ldha*), *phosphoglycerate kinase 1* (*Pgk1*), *solute carrier family 2, member 1* (*Slc2a1*), and *vascular endothelial growth factor-a* (*Vegfa*) were analyzed in the hearts of mice. N = 3–4 per group. Transcript levels were normalized to *Glucuronidase beta* (*Gusb*). Results are expressed as mean ± SD. Two-way Analysis of Variance (ANOVA) and Holm-Šidák's multiple comparisons test were used to compare the differences between groups. **(E)** Quantitative analysis and representative images of immunohistochemistry of hypoxia inducible factor-1α (HIF-1α) following 1 week of TAC, pimozide, and dapagliflozin treatment. A total of 20 slides per group from 4 mice per group were used for analysis. Results are expressed as mean ± SD. Scale bar = 50 μm. The Student's t-test was used to compare the difference between the two groups. **(F)** Scheme of our hypothesis. Dapagliflozin administration accelerates β-hydroxybutyrate oxidation and increases intracellular succinate, resulting in the activation of HIF-1α signaling.

To test this hypothesis, we examined the levels of succinate and α-ketoglutarate in the results of metabolome analysis and found no difference between the vehicle and dapagliflozin-treated groups (Supplementary Fig. 10). We also measured the abundances of succinate and α-ketoglutarate in the cardiac mitochondria and found that succinate in the mitochondrial fraction increased significantly in dapagliflozin-administered wild-type mice (Fig. 4A). The abundance of α-ketoglutarate remained unchanged (Fig. 4B), leading to the elevation of the succinate/α-ketoglutarate ratio in the hearts from dapagliflozin-treated mice (Fig. 4C).

To delineate the roles of succinate in HIF-1α signaling, we administered pimozide, a SCOT inhibitor, to TAC-operated mice. Administration of pimozide abolished the dapagliflozin-induced upregulation of HIF-1α target genes (Fig. 4D–Supplementary Table 2), as well as the accumulation of succinate in the murine heart (Supplementary Fig. 11). Pimozide-treated mice did not show an increase in HIF-1α-positive cardiomyocytes following dapagliflozin treatment (Fig. 4E). Although the data are not sufficient to draw a definitive conclusion because pimozide is a non-specific SCOT inhibitor, the results imply the possibility that accumulation of succinate is the key to activating the cardiomyocyte HIF-1α signaling in dapagliflozin-treated mice (Fig. 4F).

## 4 Discussion

In the present study, we showed that short-term administration of dapagliflozin activates HIF-1α signaling in cardiomyocytes. Inhibition of β-hydroxybutyrate metabolism abolished dapagliflozin-elicited HIF-1α activation, indicating the essential roles of ketone body oxidation in this reaction. The results highlight a possible role of cardiomyocyte HIF-1α signaling in the cardioprotective effects of SGLT2 inhibitors and illuminate succinate as a possible therapeutic target in heart failure.

Ketone body oxidation was increased in patients treated with an SGLT2 inhibitor,^17^, ^18^, ^27^ and is thought to serve as an adaptive response harboring potential benefit.^28^ The findings of this study indicate that β-hydroxybutyrate is not only oxidized as an energy substrate but also produces succinate, a signaling metabolite, and activates HIF-1α signaling in cardiomyocytes. Moreover, the results uncovered a possible link between ketone body oxidation and HIF-1α signaling.

Succinate is produced when HIF-PHs hydroxylate the proline residues of HIF-1α, and the accumulation of succinate is known to inhibit HIF-PHs.^26^ Succinate is also a byproduct of ketone body metabolism. The two ketone bodies, acetoacetate and β-hydroxybutyrate, are in equilibrium by a bi-directional reaction coupled with NAD^+^/NADH by β-hydroxybutyrate dehydrogenase 1 (BDH1). Enzyme SCOT transfers CoA from succinyl-CoA onto acetoacetate, thereby producing acetyl-CoA and succinate.

In the present study, administration of a non-specific SCOT inhibitor, pimozide, diminished succinate accumulation and HIF-1α activation. Because pimozide has multiple effects, including dopamine receptor antagonism, we cannot draw a definitive conclusion, but the results imply that the increase in β-hydroxybutyrate concentration leads to an increase in succinate concentration via SCOT and thereby inhibits HIF-PH. Succinate has been gaining focus as a signaling metabolite,^29^ and, although further studies with more specific inhibition of SCOT are warranted, this study adds a possibility of a novel layer to the metabolic-hypoxic crosstalk, namely, the ketone body-succinate-HIF-1 pathway.

In our study, dapagliflozin treatment did not alter the concentration of whole heart succinate but increased mitochondrial succinate. This difference in results may be explained by cardiomyocyte enrichment in the mitochondria assay. By volume, up to 13–22% of the whole heart is non-cardiomyocyte cells^30^, ^31^, ^32^. However, approximately 95% of the whole-heart mitochondria could be estimated to be cardiomyocyte-derived. This is because mitochondria have been reported to occupy 36% of cardiomyocyte cell volume,^33^ whereas only 2–6% of the volume of other cells, including endothelial cells.^34^ Therefore, metabolic analysis of the mitochondrial fraction could be more sensitive to the change in cardiac metabolites.

This is speculative, and indeed, there remains the possibility that succinate level is actually unchanged in cardiomyocyte cytosol and only in the mitochondria. To validate this, subcellular fractionation following cardiomyocyte isolation would be the best available method to date. However, cardiomyocyte isolation requires a significant amount of collagenase digestion in a warm buffer, which would substantially alter the metabolic landscape. Further methodological advances, including subcellular-resolution imaging and metabolomics, would aid our understanding of succinate dynamics at the subcellular level and its relations with HIF hydroxylation.

We focused on β-hydroxybutyrate metabolism because it has been reported that it increases in patients who were treated with SGLT2 inhibitors.^27^^,^^35^ The concentration of acetoacetate, another type of ketone body that is in equilibrium with β-hydroxybutyrate, remained unchanged following dapagliflozin treatment. This may be explained by two steps. First, the equilibrium is coupled with the NAD^+^/NADH ratio, and second, the failing heart has a low NAD^+^/NADH ratio.^36^ Therefore, there may have been a shift toward a high-β-hydroxybutyrate-low-acetoacetate state in the current study, which may have limited our ability to detect differences in acetoacetate levels. Acetone, another ketone body, is volatile and was not possible to quantify in our ion chromatography-based analysis, which is a limitation of our study. Other metabolites, including those in the inositol metabolism pathway, which also ranked high in the enrichment analysis, may also act as signaling metabolites. Although we were not able to quantify it in our metabolome analysis, inositol and its derivative, inositol triphosphate, have been reported to play a crucial role in cardiomyocyte signaling and cardiac function.^37^^,^^38^ Further studies are warranted to elucidate its role in the context of SGLT2 inhibitors and the heart.

One major limitation of the present study is that we performed metabolome analysis on the extracted water-soluble metabolites and excluded the lipid-soluble metabolites, which include fatty acids, acyl-CoAs, and acylcarnitines. Succinate can arise from several intersecting pathways, and other potential signaling metabolites could also play a role in the augmentation of HIF-1α signaling following SGLT2 treatment, which requires further study. Moreover, the relatively small number of samples included in the metabolomics analysis may have hindered the detection of alterations within the water-soluble metabolites as well. Further studies with large-scale and broad analysis of the metabolic changes following short-term SGLT2 treatment would be warranted to further understand its effect on the cardiac metabolic landscape.

Another limitation of our study is that, although we have shown that HIF-1α is accumulated and HIF-1α signaling is augmented following dapagliflozin treatment, we did not directly quantify HIF-PH activity *in vivo*. The oxygen-dependent degradation domain (ODD)-Luc mice, which express a fusion protein composed of the ODD of HIF-1α and firefly luciferase,^39^ could be one possible solution to further examine how, when, and to what extent HIF-PHs are activated following dapagliflozin treatment. Quantification of hydroxylated HIF-1α could be another option, while the specificity of the method in detecting *in vivo* changes in cardiomyocytes could be a challenge.

While there is no direct evidence that HIF-1 is activated in human patients receiving SGLT2 inhibitor treatment, several studies indirectly suggest the possibility. VEGF-A is a downstream target of HIF-1α, and it has been reported to induce upregulation of circulating endothelial progenitor cells in human subjects.^40^ Circulating endothelial cells have been reported to increase in human patients receiving SGLT2 inhibitors,^41^ which may also be a consequence of upregulated HIF-1α signaling. Additionally, in a porcine model of chronic myocardial ischemia, HIF-1α has been reported to augment myocardial perfusion during stress,^42^ and dapagliflozin has been reported to improve myocardial flow reserve in a small human study.^43^ These results are indirect evidence and are still inconclusive about whether SGLT2 inhibitors upregulate HIF-1α in human subjects. Further study is warranted to address the knowledge gap.

The relevance of our results to the cardioprotective role of SGLT2 inhibitors remains unclear, and our results need careful interpretation regarding their functional significance. In our study, dapagliflozin had no significant functional benefit in the pressure-overloaded heart, but meanwhile, it augmented HIF-1α signaling. This disconnect implies three possibilities: First, the effect of HIF-1α signaling may vary depending on the context. For example, cardiomyocyte-specific inducible HIF-1 knockout mice using α*MHC-MerCreMer* showed worsened cardiac function upon pressure overload,^14^ while non-inducible cardiomyocyte-specific HIF-1α knockout mice using *Mlc2v-Cre* showed improvement of heart failure.^44^ Whether the HIF-1α signal is beneficial or not may vary depending on the timing of HIF-1α activation and the background condition of the heart, which needs further evaluation. Second, even HIF-1α itself is beneficial, the extent of the HIF-1α signal may not have been enough. The upregulation of HIF-1α target genes was significant, but the increase in expression was 1.3–1.5 fold. This may not have been enough to convey the beneficial effects. Moreover, the number of samples was relatively small, and there remains a possibility that the actual fold change could be even lower. Third, the benefits of HIF-1α may depend on the mode of activation. In the current study, dapagliflozin administration did not increase the expression of all HIF-1α target genes in the heart. HIF-1α is stabilized in various conditions, including hypoxia and toll-like receptor activation; however, the HIF-1α target genes upregulated in each condition differ.^45^ Upregulation of HIF-1α signaling by dapagliflozin may have similar characteristics, accounting for the partial activation of HIF-1α target genes and the lack of improvement in cardiac function. The activation mechanisms of HIF-1α and its effect on cardiac function could be complex, and further studies are needed to determine how, if any, dapagliflozin-induced activation of HIF-1α signaling plays a beneficial role in cardiac function.

This uncertainty is further underscored by discrepancies between our findings and previous animal studies. Several studies have reported the cardioprotective effects of SGLT2 inhibitors in murine models of pressure-overload-induced heart failure,^46^^,^^47^ and the reason we did not see the functional improvement is unclear. There are several possible reasons, including limited animal numbers in the study, differences in the observational period, duration of SGLT2 inhibitor treatment, severity of TAC, and distinct SGLT2 inhibitors used in the animal studies. There may be underlying molecular explanations warranting further studies, which could lead to a better understanding of the difference between responders and non-responders against SGLT2 inhibitors. A relatively low number of samples included in the study may also have decreased the sensitivity of the functional analysis.

In addition to functional outcomes, our data raise questions regarding tissue remodeling in the heart. In the present study, after the exclusion of an outlier, there was a significant difference in the cardiac fibrosis between vehicle-treated control mice and vehicle-treated cmHIF-1α CKO mice. Two points should be noted regarding this result. First, the outlier-removed data should be considered as a reference because we have decided to perform outlier removal after we collected and observed the original data. What the outlier removal implies is the mere possibility that cardiomyocyte HIF-1α could increase cardiac fibrosis upon acute pressure overload, and the need for further study regarding the relationship. Second, if the relationship after the outlier removal better reflects the actual relationship, this may indicate that cardiomyocyte HIF-1α promotes fibroblast activation and promotes tissue fibrosis. Because HIF-1α is a key transcription factor that modulates the balance between oxidative phosphorylation and glycolysis, its activation in cardiomyocytes could lead to increased lactate production and low pH in the tissue microenvironment, which then could activate fibroblasts and promote tissue remodeling.^48^^,^^49^ This effect could be cancelled by the administration of SGLT2 inhibitors by changing the metabolism of both cardiomyocytes and fibroblasts. This interpretation is still speculative, and further studies are needed to elucidate the relationship between cardiomyocyte HIF-1α, tissue fibrosis, and SGLT2 inhibitors.

These observations also prompt consideration of the temporal dynamics of HIF-1α signaling. The current study focused on the acute phase of pressure overload; however, the extent to which the results of this study can be extrapolated to a more long-term chronic heart failure remains unknown. Upregulation of cardiomyocyte HIF-1α was no longer observed in the heart following 4 weeks of dapagliflozin treatment in the present study. The underlying mechanisms are unknown, but it may be due to the complex and still controversial nature of cardiomyocyte HIF-1α signaling following long-term pressure overload. Some animal studies show that following 4 weeks of pressure overload, cardiomyocyte HIF-1α signal attenuates,^14^ whereas patients with hypertrophic cardiomyopathy, which is a chronic disease, have upregulated HIF-1α.^44^ One key to understanding this controversy may be the negative feedback loop of HIF-1α. HIF-1α has been reported to directly activate the transcription of prolyl hydroxylase domain 2 (PHD2), which degrades HIF-1α.^50^ Continuous activation of HIF-1α can be hindered by this negative feedback. Long-term HIF-1α activation is a more complex process compared to short-term activation, and the results of the present study may not fully represent the critical mechanisms underlying the cardioprotective effect of dapagliflozin treatment in chronic heart failure patients.

These findings suggest broader implications for cardiac metabolic regulation. The heart is metabolically an omnivorous organ, and utilizes multiple energy substrates including fatty acids, glucose, amino acids, and ketone bodies. Ketone body oxidation, however, is known to suppress glucose uptake and oxidation in cardiomyocytes.^51^, ^52^, ^53^ While evidence suggests that increasing ketone body oxidation is beneficial in the murine models of heart failure,^54^, ^55^, ^56^ excessive reliance on a specific energy source may impair the heart's metabolic flexibility.^57^^,^^58^ Drug-elicited activation of HIF-1α signaling may, in the long term, restore glucose utilization by upregulating the molecules involved in glucose utilization and reinforce the heart's metabolic flexibility.

In summary, our results suggest a novel function of SGLT2 inhibitors activating HIF-1α signaling, which is a critical transcription factor for the regulation of angiogenesis, metabolic adaptation, and response to hypoxia. This effect was mediated via metabolic signaling. SGLT2 inhibitors have been reported to exert multiple beneficial effects on various organs, including the heart, kidney, and liver^1^, ^2^, ^3^^,^^59^^,^^60^. However, the underlying mechanisms of their effects remain unknown. Our results suggest that ketone bodies and succinate are metabolic signals that mediate the potential mechanisms of SGLT2 inhibitors. These results provide a foundation for future research and development of novel therapeutics.

## CRediT authorship contribution statement

**Tatsuyuki Sato:** Writing – review & editing, Writing – original draft, Visualization, Investigation, Formal analysis, Data curation, Conceptualization. **Takayuki Isagawa:** Writing – review & editing, Project administration, Investigation. **Yuki Sugiura:** Writing – review & editing, Formal analysis, Data curation. **Daigo Sawaki:** Writing – review & editing. **Yu Nakagama:** Writing – review & editing. **Takahiro Kuchimaru:** Writing – review & editing. **Shun Minatsuki:** Writing – review & editing. **Shigeru Sato:** Writing – review & editing. **Kazutoshi Ono:** Writing – review & editing. **Ariunbold Chuluun-Erdene:** Writing – review & editing. **Hiroaki Semba:** Writing – review & editing. **Masamichi Ito:** Writing – review & editing. **Toshinaru Kawakami:** Writing – review & editing. **Ryohei Tanaka:** Writing – review & editing. **Masaya Sakamoto:** Writing – review & editing. **Masataka Asagiri:** Writing – review & editing. **Hiroshi Harada:** Writing – review & editing. **Christian Stockmann:** Writing – review & editing. **Tomo Yonezawa:** Writing – review & editing. **Yasushi Hirota:** Writing – review & editing. **Yasutoshi Kido:** Writing – review & editing. **Kinya Otsu:** Resources. **Takahide Kohro:** Writing – review & editing. **Ichiro Manabe:** Writing – review & editing, Data curation. **Issei Komuro:** Writing – review & editing. **Norihiko Takeda:** Writing – review & editing, Writing – original draft, Supervision, Project administration, Funding acquisition, Conceptualization.

## Disclosures

T.S. has received grant support from The Nakatomi Foundation, The Japanese Heart Failure Society, Fukuda Foundation for Medical Technology, and The Cell Science Research Foundation, and has received honoraria from Sunrise Lab. N.T. has received grant support from Daiichi Sankyo Company. Ltd, Bayer Yakuhin, Ltd, AstraZeneca K.K, Ono Pharmaceutical Co., Ltd, and Bristol Myers Squibb. I.K. has received grant support from Daiichi Sankyo Company, Ltd, Idorsia Pharmaceuticals Japan Ltd, Takeda Pharmaceutical Company Ltd., Mitsubishi Tanabe Pharma Corporation, and TEIJIN PHARMA LIMITED., and has received honoraria from ONO PHARMACEUTICAL CO. LTD., AstraZeneca K.K., and Nippon Boehringer Ingelheim Co. Ltd. Y.N. and Y.K. have received joint research funding from ROHTO Pharmaceutical Co., Ltd. The other authors declare no competing interests.
