# Supplementary material for: Sodium-glucose Co-transporter 2 (SGLT2) inhibitor dapagliflozin acutely activates cardiomyocyte HIF-1α signaling via succinate, a signaling metabolite
Source: J Pharmacol Sci. 2026 Apr;160(4):221–31. doi: 10.1016/j.jphs.2026.01.008 (PMC12979004; doi:10.1016/j.jphs.2026.01.008)
Supplement: Multimedia component 1 [file mmc1.pdf]

Supplementary Method

Tissue sampling and metabolome analysis by ion chromatography (IC)-mass spectrometry (MS)

We anesthetized the mice with isoflurane and euthanized the mice with a laboratory microwave instrument (MMW-05; Muromachi Kikai, Tokyo, Japan) to instantly inactivate metabolic enzymes within the heart tissue. The heart was sampled and homogenized together with internal control compounds (20 μM each of L-Methionine sulfone, 2-Morpholinoethanesulfonic acid, monohydrate [MES], and D-Camphor-10-sulfonic Acid Sodium Salt [CSA]) in ice-cold methanol (500μL) using a manual homogenizer (Finger Masher, AM79330; Sarstedt, Tokyo, Japan). We then added an equal volume of chloroform and 0.4 times the volume of ultrapure water. The suspension was centrifuged at 4600 g for 20 min at 4ºC, and the aqueous phase was ultrafiltrated with an ultrafiltration tube (Ultrafree-MC, UFC3 LCC NB; Human Metabolome Technologies, Tsuruoka, Japan). The filtrate was concentrated using a vacuum concentrator (PV-1200; WakenBtech, Kyoto, Japan). The concentrated filtrate was dissolved in 150 μL of ultrapure water containing internal control compounds (3-Aminopyrrolidine and Trimesate) and used for IC-MS.

Anionic metabolites were measured using an Orbitrap-type MS (Q-Exactive focus; Thermo Fisher Scientific) connected to a high-performance IC system (ICS-5000+, Thermo Fisher Scientific) that utilizes the IC-separation and Fourier Transfer MS principle and enables us to perform highly selective and sensitive quantification of glycolytic metabolites, tricarboxylic acid cycle intermediates, as well as ketone bodies. An anion electrolytic suppressor (Thermo Scientific Dionex AERS 500) was used to convert the potassium hydroxide gradient into pure water before the sample entered the mass spectrometer. The Thermo Scientific Dionex IonPac AS11-HC, 4-µm particle size column, was used in the IC system. The IC flow rate was 0.25 mL/min supplemented post-column with 0.18 mL/min makeup flow of MeOH. The following conditions were used in the potassium hydroxide gradient of the IC system: from 1 mM to 100 mM (0–40 min), 100 mM (40–50 min), and 1 mM (50.1–60 min) at a column temperature of 30°C. The Q Exactive focus mass spectrometer was operated under an ESI negative mode for all detections. Full mass scan (m/z 70−900) with a resolution of 70,000 was performed. The automatic gain control target was set at 3×10^6 ions, and the maximum ion injection time was 100 msec. Source ionization parameters were optimized with the spray voltage at 3 kV, and other parameters were as follows: transfer temperature = 320°C, S-Lens level = 50, heater temperature = 300°C, Sheath gas = 36, and Aux gas = 10.

We used Compound Discoverer 3.1 (Thermo Fisher Scientific) for the non-targeted metabolomics workflow. In brief, this software first aligned the total ion chromatograms of different samples along the retention time. Then, the detected features with an intensity of no less than 100,000 and an S/N larger than 5 in each set of data were extracted and merged into components. Each compound was identified based on (i) formula prediction based on accurate m/z value and isotope peak patterns and (ii) MS/MS structural validation. Formula predicted signals were also assigned into candidate compounds by database search (Chemspider database; http://www.chemspider.com/). Volcano plotting based on a T-test was performed and presented. Enrichment analyses were performed using MetaboAnalyst (v6.0), a web-based multivariate analysis tool.[63] For normalization, samples were subjected to median adjustment to uniformly correct for systematic differences across samples. Autoscaling was applied to standardize comparisons of variables, without performing data transformation.

Supplementary Table 1. Numerical fold changes of hypoxia inducible factor-1α (HIF-1α) target genes relative to vehicle-treated control mice in Figures 2A and 2B.

	Control-Dapagliflozin	KO-Vehicle	KO-Dapagliflozin
Ldha	1.32078630	0.75789754	0.78180333
Vegfa	1.41890154	0.78621098	0.98003710
Glut1	1.36360313	0.70408034	0.78340823
Pgk1	1.16389854	0.84951284	0.80945458

Supplementary Table 2. Numerical fold changes of hypoxia inducible factor-1α (HIF-1α) target genes relative to vehicle-treated mice in Figure 4D.

	Dapagliflozin
Ldha	1.13846253
Vegfa	1.22984772
Glut1	1.05512569
Pgk1	1.00296062

Supplementary Figure Legends

Figure S1. Analysis of the fibrotic area following the exclusion of an outlier

Data show the analysis of cardiac fibrosis of hearts sampled after 1 week of dapagliflozin administration in control and cardiomyocyte-specific HIF-1α knockout (cmHIF-1α CKO) mice. One datapoint was excluded following robust regression and outlier removal (ROUT) method. Results are expressed as mean ± SD. N = 4–9 per group. Two-way Analysis of Variance (ANOVA) and *Holm-Šidák's* multiple comparisons test were used to compare the differences between groups.

Figure S2. The peak pressure gradient of TAC in control and cmHIF-1α CKO mice

The peak pressure gradient of transverse aortic constriction (TAC) was measured using pulsed wave Doppler at the point of constriction in control and cardiomyocyte-specific hypoxia inducible factor-1α knockout (cmHIF-1α CKO) mice. Results are expressed as mean ± SD. N = 2–3 per group. Two-way Analysis of Variance (ANOVA) and *Holm-Šidák's* multiple comparisons test were used to compare the differences between groups.

Figure S3. The knockout efficiency of cmHIF-1α CKO mice

The knockout efficiency of hypoxia inducible factor-1α (HIF-1α) was evaluated using a primer set that includes exon 2 of the *Hif1a* gene and a primer set that does not include exon 2 of the *Hif1a* gene. Results are expressed as mean ± SD. N = 3–8 per group.

Figure S4. The effect of dapagliflozin on HIF-1α signaling 4 weeks after pressure-overload and dapagliflozin treatment

(A, B) Transcript levels of HIF-1α target genes *lactate dehydrogenase A* (*Ldha*), *phosphoglycerate kinase 1* (*Pgk1*), *solute carrier family 2, member 1* (*Slc2a1*), and *vascular endothelial growth factor-a* (*Vegfa*) were analyzed in the hearts sampled after 4 weeks of dapagliflozin administration in control and cardiomyocyte-specific hypoxia inducible factor-1α knockout (cmHIF-1α CKO) mice. N = 3–4 per group. Transcript levels were normalized to *Glucuronidase beta* (*Gusb*). Results are expressed as mean ± SD. Two-way Analysis of Variance (ANOVA) and *Holm-Šidák's* multiple comparisons test were used to compare the differences between groups.

Figure S5. The effect of dapagliflozin on HIF-PH expression

(A, B) Transcript levels of hypoxia-inducible factor-prolyl hydroxylases (HIF-PHs), *prolyl hydroxylase domain 1* (*Phd1*), *prolyl hydroxylase domain 2* (*Phd2*), and *prolyl hydroxylase domain 3* (*Phd3*), were analyzed in the hearts sampled after 1 week of dapagliflozin administration in control mice. N = 7–8 per group. Transcript levels were normalized to *Glucuronidase beta* (*Gusb*). Results are expressed as mean ± SD. Two-way Analysis of Variance (ANOVA) and *Holm-Šidák's* multiple comparisons test were used to compare the differences between groups.

Figure S6–9. Quantitative analysis of individual metabolites from metabolome analysis

Individual metabolites were quantified from the metabolome analysis of hydrophilic metabolites extracted from the whole heart following 1 week of dapagliflozin or vehicle administration. Each point represents the result for an individual mouse. N = 5 per group. Results are expressed as mean ± SD. The Student's t-test was used to compare the differences between the two groups.

Figure S10. Quantification of succinate and α-ketoglutarate levels in the whole heart extract

(A–C) Succinate and α-ketoglutarate levels were quantified in hydrophilic metabolites extracted from the hearts following 1 week of dapagliflozin or vehicle administration. N = 5–6 per group. Results are expressed as mean ± SD. The Student's t-test was used to compare the difference between the two groups.

Figure S11. Dapagliflozin does not induce succinate accumulation in pimozide-treated hearts

Succinate and α-ketoglutarate levels in the mitochondrial fraction of cardiac tissues were quantified 1 week after dapagliflozin and pimozide or vehicle and pimozide administration. N = 5–6 per group. Results are expressed as mean ± SD. The Student's t-test was used to compare the difference between the two groups.

Fig. S1

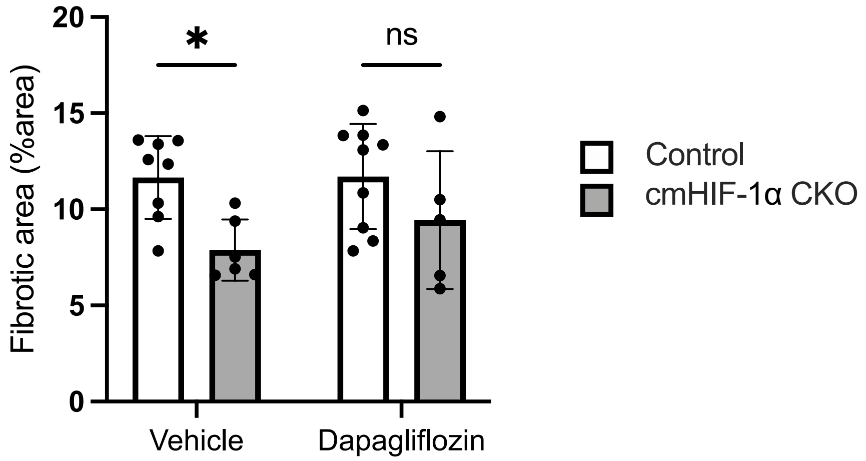

Fibrotic area (%area)
20
15
10
5
0
*
ns
Vehicle
Dapagliflozin
Control
cmHIF-1α CKO

Fig. S2

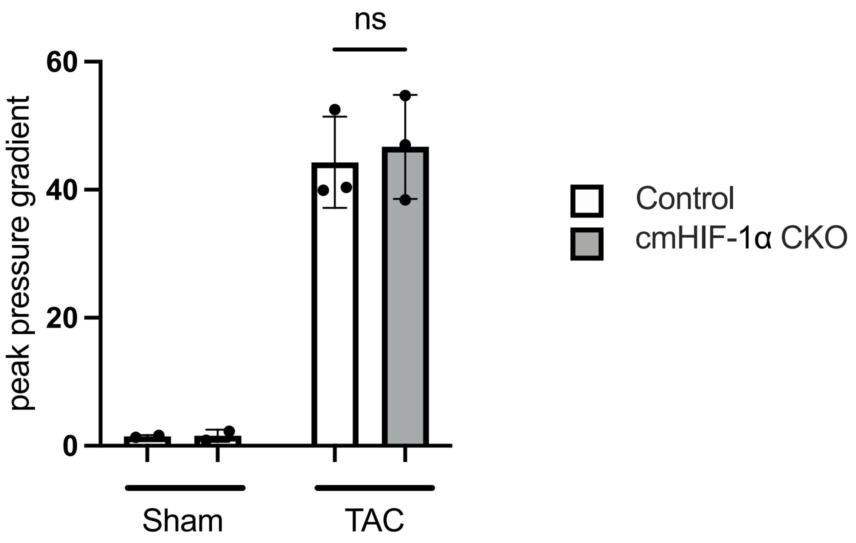

ns
60
40
20
0
peak pressure gradient
Sham
TAC
Control
cmHIF-1α CKO

Fig. S3

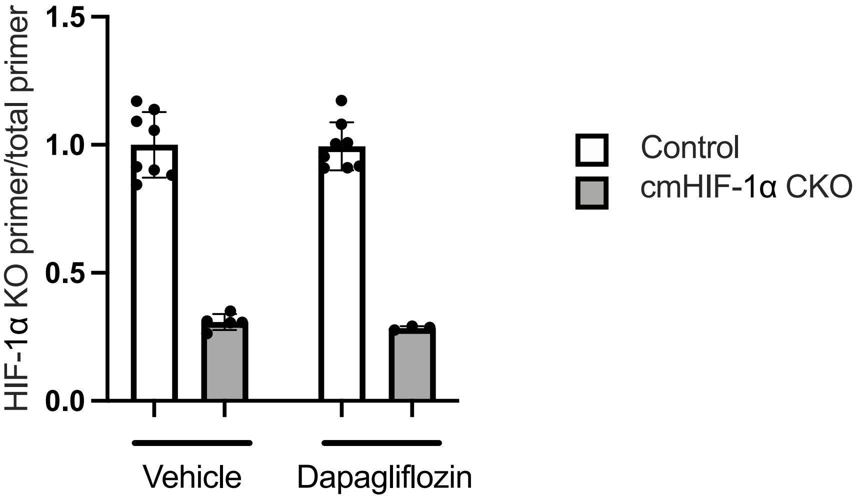
HIF-1α KO primer/total primer
1.5
1.0
0.5
0.0
Vehicle
Dapagliflozin
Control
cmHIF-1α CKO

Fig. S4

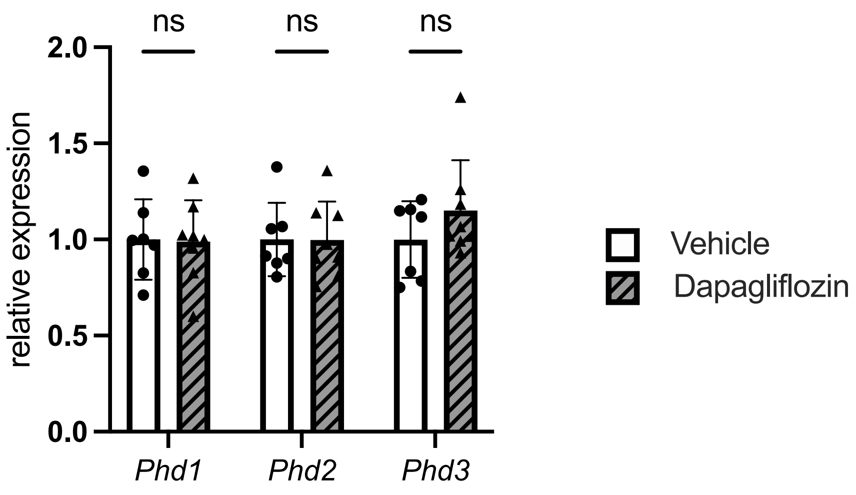

p = 0.6312 for drug

Fig. S5

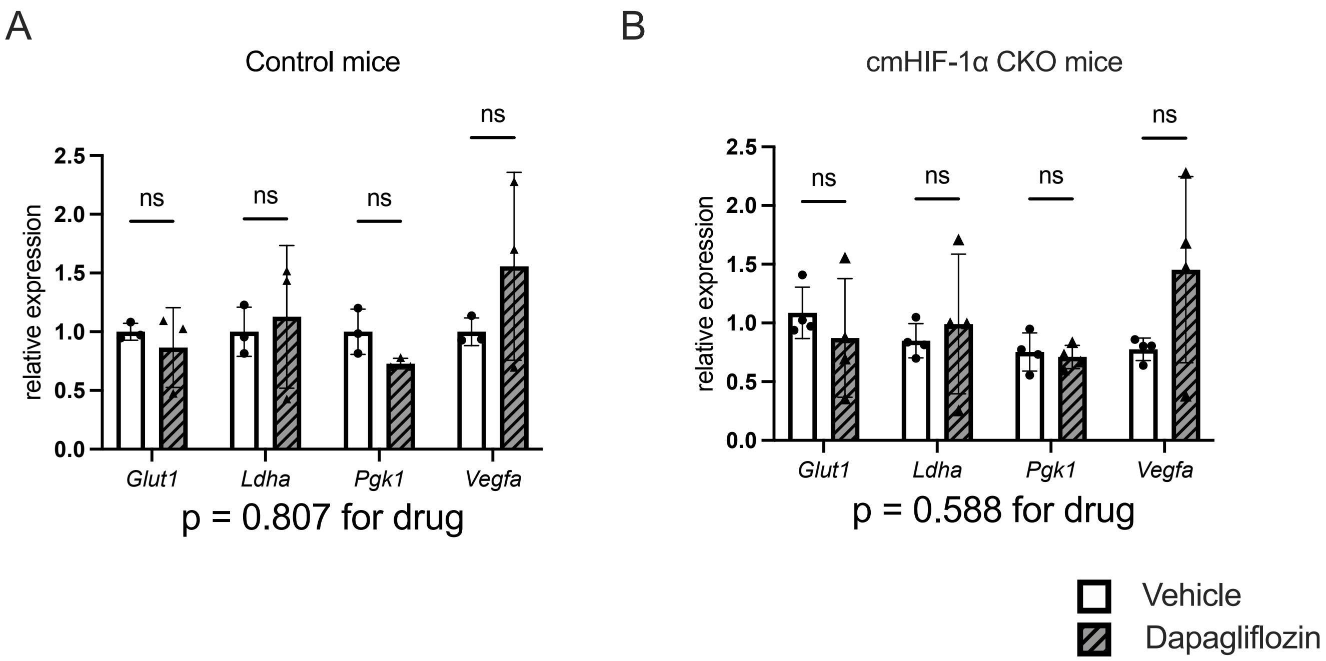

A
Control mice
ns
relative expression
2.5
2.0
1.5
1.0
0.5
0.0
Glut1
Ldha
Pgk1
Vegfa
p = 0.807 for drug
B
cmHIF-1α CKO mice
p = 0.588 for drug
Vehicle
Dapagliflozin

Fig. S6

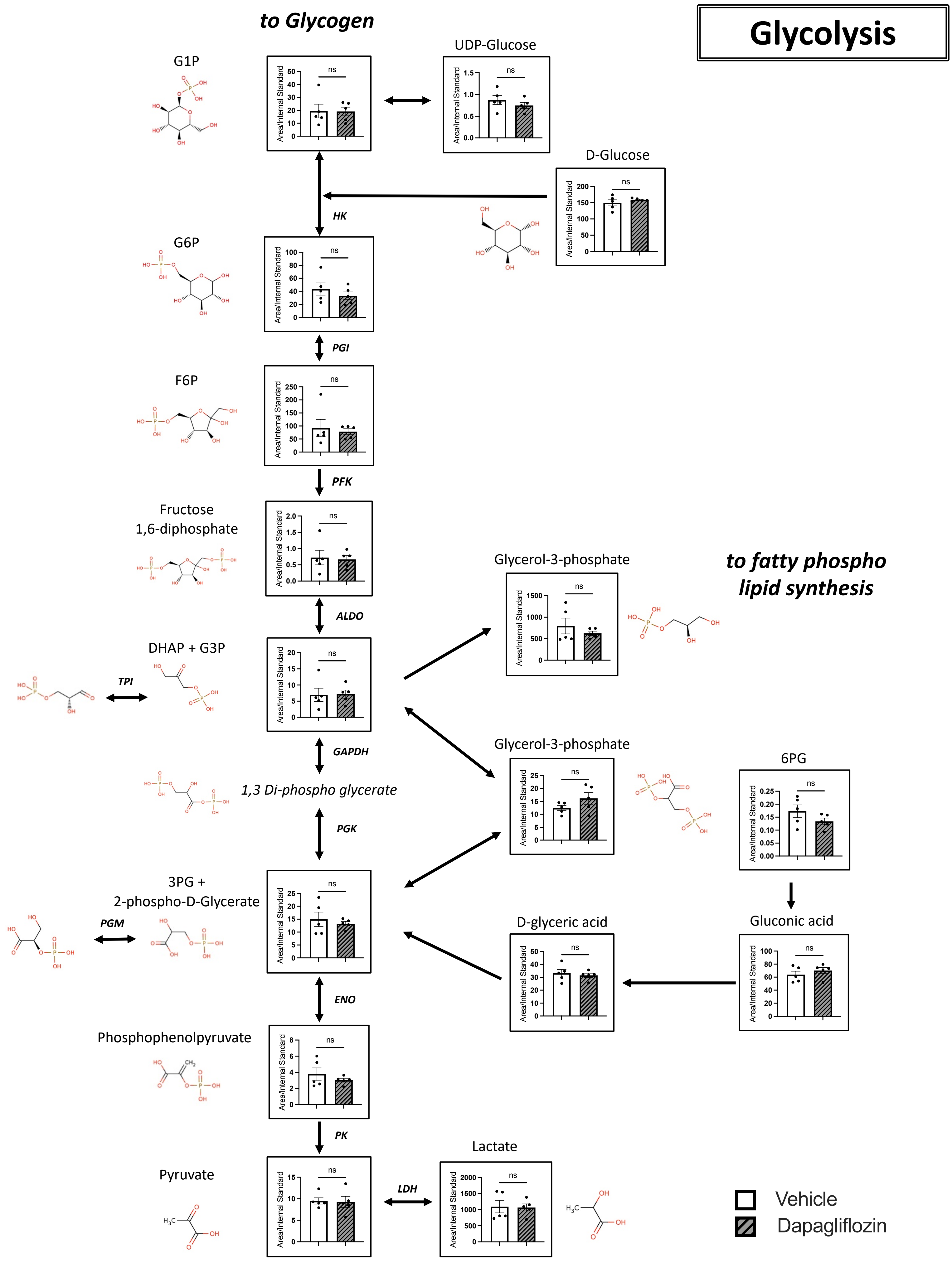

Glycolysis
to Glycogen
G1P
UDP-Glucose
D-Glucose
HK
G6P
PGI
F6P
PFK
Fructose 1,6-diphosphate
ALDO
Glycerol-3-phosphate
to fatty phospho lipid synthesis
DHAP + G3P
TPI
GAPDH
1,3 Di-phospho glycerate
Glycerol-3-phosphate
6PG
PGK
3PG + 2-phospho-D-Glycerate
PGM
D-glyceric acid
Gluconic acid
ENO
Phosphophenolpyruvate
PK
Pyruvate
LDH
Lactate
Area/Internal Standard
ns
Vehicle
Dapagliflozin

Fig. S7

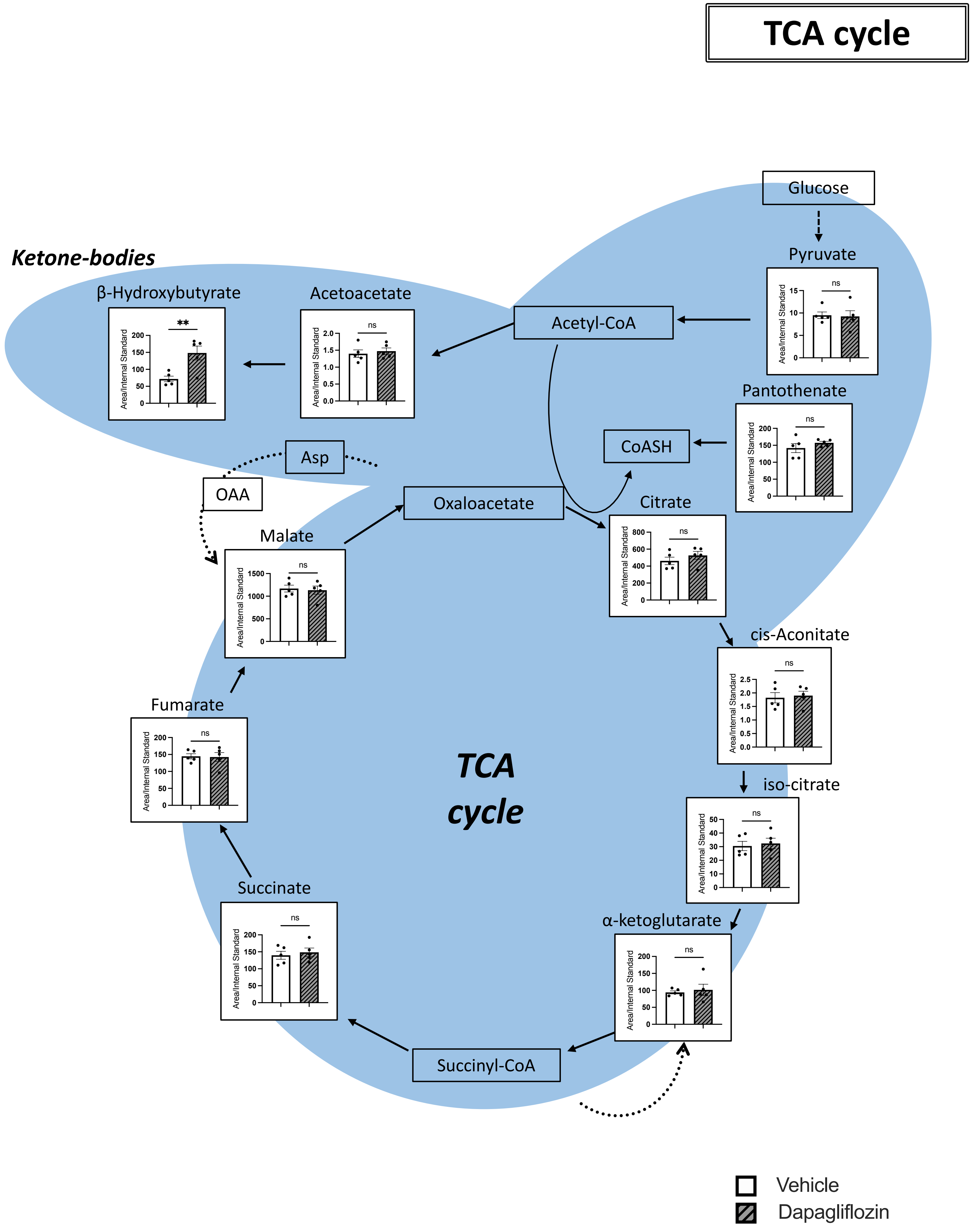
TCA cycle
Glucose
Pyruvate
Acetyl-CoA
Ketone-bodies
β-Hydroxybutyrate
Acetoacetate
Pantothenate
CoASH
Asp
OAA
Oxaloacetate
Citrate
Malate
cis-Aconitate
iso-citrate
Fumarate
TCA cycle
Succinate
α-ketoglutarate
Succinyl-CoA
Area/Internal Standard
ns
**
Vehicle
Dapagliflozin

Fig. S8

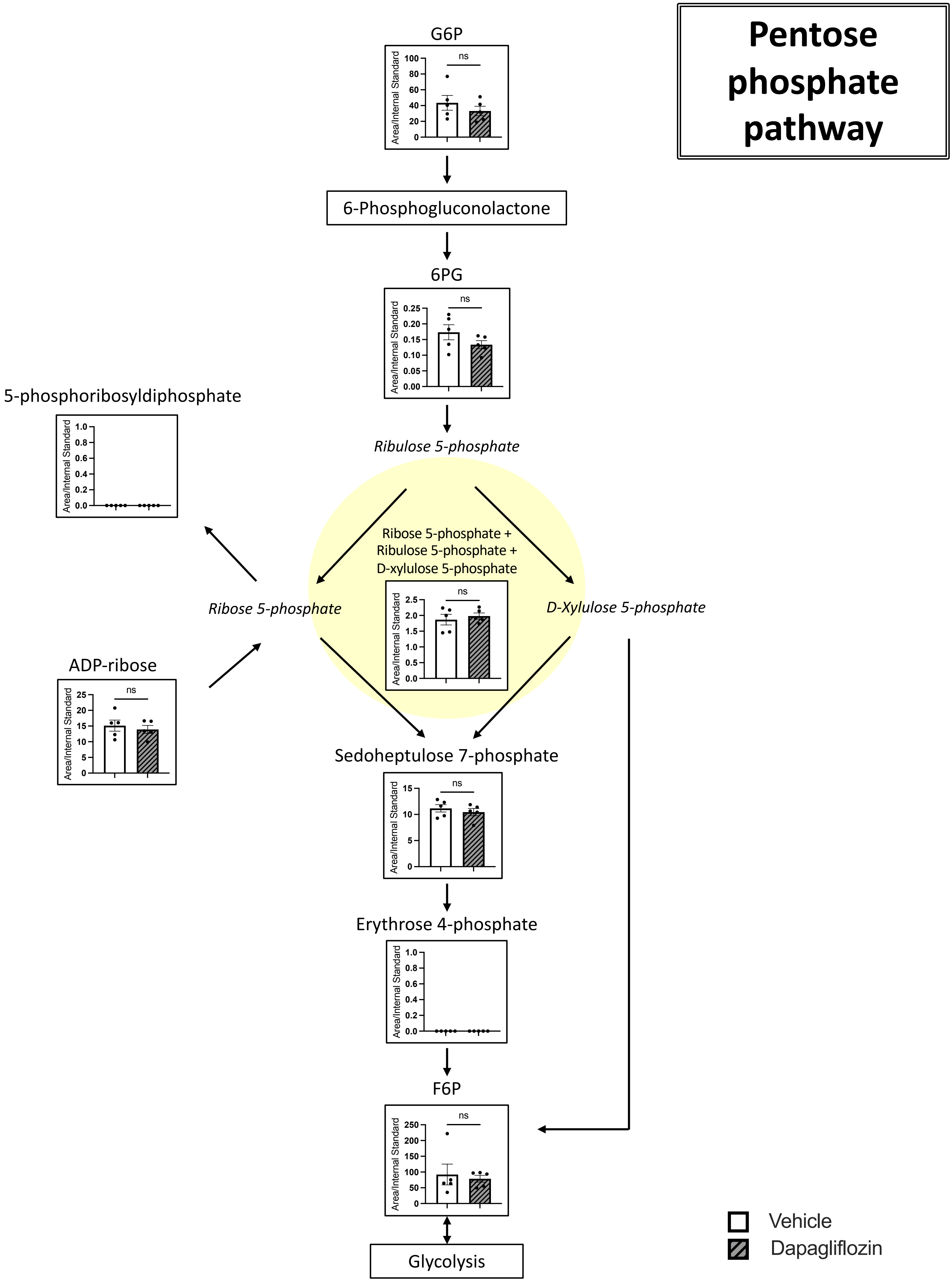
Pentose phosphate pathway
G6P
6-Phosphogluconolactone
6PG
Ribulose 5-phosphate
5-phosphoribosyldiphosphate
Ribose 5-phosphate + Ribulose 5-phosphate + D-xylulose 5-phosphate
Ribose 5-phosphate
D-Xylulose 5-phosphate
ADP-ribose
Sedoheptulose 7-phosphate
Erythrose 4-phosphate
F6P
Glycolysis
Area/Internal Standard
ns
Vehicle
Dapagliflozin

Fig. S9

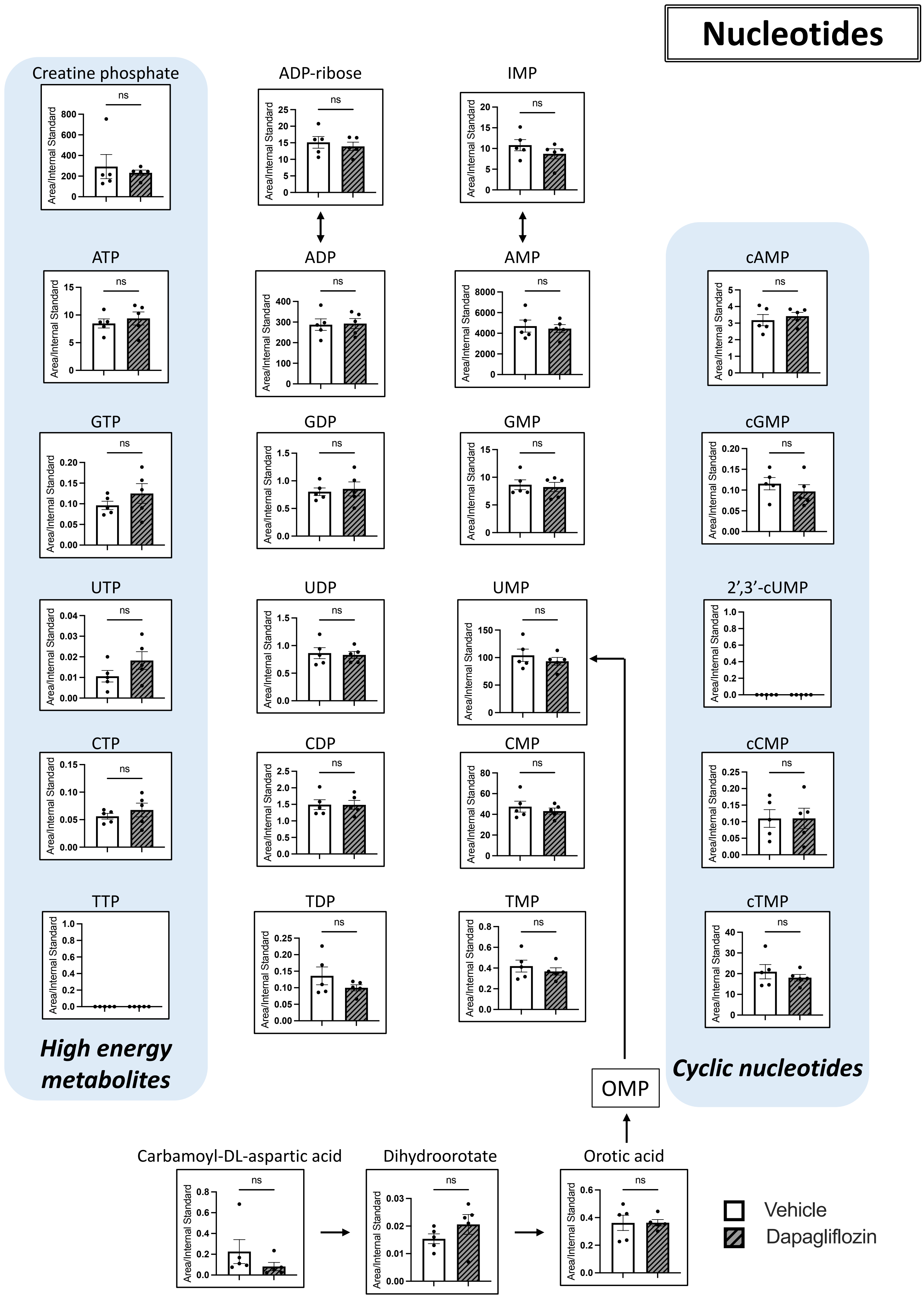
Nucleotides
Creatine phosphate
ADP-ribose
IMP
ATP
ADP
AMP
cAMP
GTP
GDP
GMP
cGMP
UTP
UDP
UMP
2’,3’-cUMP
CTP
CDP
CMP
cCMP
TTP
TDP
TMP
cTMP
High energy metabolites
Cyclic nucleotides
OMP
Carbamoyl-DL-aspartic acid
Dihydroorotate
Orotic acid
Area/Internal Standard
ns
Vehicle
Dapagliflozin

Fig. S10

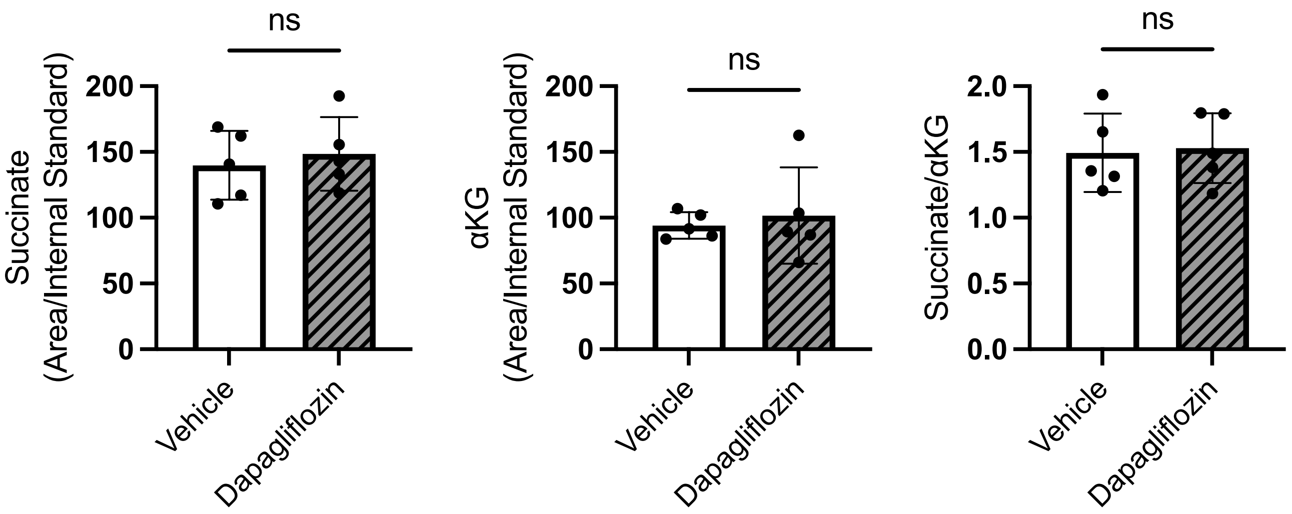

ns
Succinate
(Area/Internal Standard)
200
150
100
50
0
Vehicle
Dapagliflozin
ns
αKG
(Area/Internal Standard)
200
150
100
50
0
Vehicle
Dapagliflozin
ns
Succinate/αKG
2.0
1.5
1.0
0.5
0.0
Vehicle
Dapagliflozin

Fig. S11

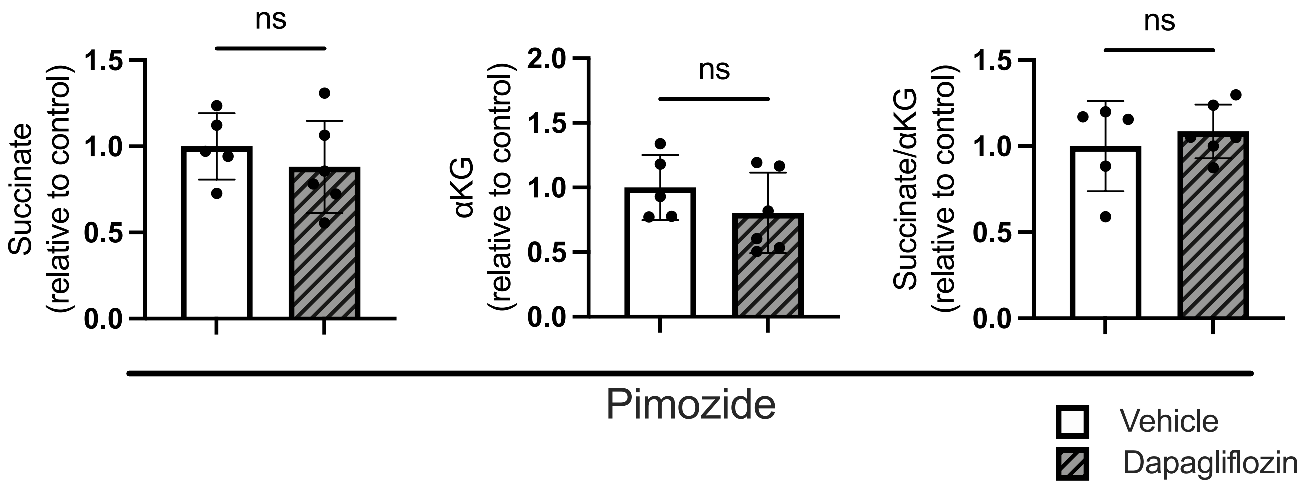
ns
Succinate
(relative to control)
1.5
1.0
0.5
0.0
ns
αKG
(relative to control)
2.0
1.5
1.0
0.5
0.0
ns
Succinate/αKG
(relative to control)
1.5
1.0
0.5
0.0
Pimozide
Vehicle
Dapagliflozin
